# Evaluation of real-time PCR assay to detect *Schistosoma mansoni* infections in a low endemic setting

**DOI:** 10.1186/s12879-014-0558-4

**Published:** 2014-10-23

**Authors:** Maria Cristina Carvalho Espírito-Santo, Mónica Viviana Alvarado-Mora, Emmanuel Dias-Neto, Lívia Souza Botelho-Lima, João Paulo Moreira, Maria Amorim, Pedro Luiz Silva Pinto, Ashley R Heath, Vera Lúcia Pagliusi Castilho, Elenice Messias do Nascimento Gonçalves, Expedito José de Albuquerque Luna, Flair José Carrilho, João Renato Rebello Pinho, Ronaldo Cesar Borges Gryschek

**Affiliations:** Department of Infectious and Parasitic Diseases, School of Medicine of the University of São Paulo, LIM-06 São Paulo, SP Brazil; Laboratory of Tropical Gastroenterology and Hepatology, Department of Gastroenterology, School of Medicine of the University of São Paulo, São Paulo, SP Brazil; Laboratory of Medical Genomics, AC Camargo Cancer Center, São Paulo, SP Brazil; Department of Enteroparasites at the Parasitology and Mycology Service from the Adolfo Lutz Institute, São Paulo, SP Brazil; Institute of Tropical Medicine, University of São Paulo, São Paulo, SP Brazil; Parasitology Section of the Central Laboratory Division, Hospital das Clínicas, School of Medicine of the University of São Paulo, São Paulo, SP Brazil; Sigma Custom Products, 9186 Six Pines Drive, The Woodlands, 77380 TX USA; Laboratory of Neurosciences Alzira Denise Hertzog Silva (LIM-27) Institute and Department of Psychiatry, University of São Paulo, São Paulo, SP Brazil; University Center of Volta Redonda, State of Rio de Janeiro, Brazil

**Keywords:** Schistosomiasis mansoni, TaqMan® Real-Time PCR system, Laboratory diagnosis, Low endemicity areas, Brazil/epidemiology

## Abstract

**Background:**

Schistosomiasis constitutes a major public health problem, and 200 million people are estimated to be infected with schistosomiasis worldwide. In Brazil, schistosomiasis has been reported in 19 states, showing areas of high and medium endemicity and a wide range of areas of low endemicity (ALE). Barra Mansa in Rio de Janeiro state has an estimated prevalence of 1%. ALE represent a new challenge for the helminth control because about 75% of infected individuals are asymptomatic and infections occur with a low parasite load (<100 eggs per gram of feces), causing a decrease in sensitivity of stool parasitological techniques, which are a reference for the laboratory diagnosis of this helminth. The objective of this study was to evaluate the performance of a TaqMan quantitative polymerase chain reaction (qPCR) technique in serum and feces DNA samples using the techniques of Kato-Katz (KK), Hoffman, Pons and Janer (HH) as references, during an epidemiological survey using fecal samples and sera from randomized residents from an ALE.

**Methods:**

A cross-sectional study conducted from April to December 2011 using a probabilistic sampling that collected 572 fecal and serum samples. The laboratory diagnostic techniques used were: KK, HH and qPCR (feces and serum).

**Results:**

We obtained the following results using the different diagnostic techniques: KK and HH, 0.9% (n =5); qPCR-feces, 9.6% (n =55); and qPCR-serum, 1.4% (n =8). The qPCR-feces presented the highest positivity, whereas the techniques of HH and KK were the least sensitive to detect infections (0.8%). Compared to HH and KK, qPCR-feces showed a statistically significant difference in positivity (*p* <0.05), although with poor agreement.

**Conclusion:**

The positivity rate presented by the qPCR approach was far higher than that obtained by parasitological techniques. The lack of adequate surveillance in ALE of schistosomiasis indicates a high possibility of these areas being actually of medium and high endemicity. This study presents a control perspective, pointing to the possibility of using combined laboratory tools in the diagnosis of schistosomiasis in ALE.

**Electronic supplementary material:**

The online version of this article (doi:10.1186/s12879-014-0558-4) contains supplementary material, which is available to authorized users.

## Background

Schistosomiasis constitutes a major public health problem, and 200 million people around the world are estimated to be infected [1],[2]. In Brazil, the infection has been reported in 19 states [3], including areas of high, medium, and low endemicity. Among the several known *Schistosoma* species, *S. mansoni* represents the most prevalent species around the world and it is the only causative agent of schistosomiasis in Brazil [4].

Although severe forms of the disease have been less frequently reported mostly due to the use of mass chemotherapy, schistosomiasis continues to spread mainly in connection with the extension of agricultural zones and irrigated areas [5]. In areas of low endemicity, 80% of the individuals are asymptomatic or present only the mild form of the disease, which hinders the diagnosis of the infection [6].

One of the focal areas of schistosomiasis due to *S. mansoni* in Rio de Janeiro State is located in the municipality of Barra Mansa [7], which has an estimated prevalence of 1%. The endemic areas are within the urban perimeter, with a higher prevalence observed in the neighborhood of Siderlândia, followed by the neighborhoods of Santa Clara, São Luiz, Cantagalo, and Nova Esperança [8].

Measures to control this endemic disease in the municipality were first taken in 1976 with the implementation of the Special Schistosomiasis Control Program (PECE) [9]. The parasitological techniques of Hoffman and Kato-Katz are used for the diagnosis of this helminthic infection.

Stool parasitological techniques have been historically used as a reference to the laboratory diagnosis of schistosomiasis. Although these techniques are low-cost and simple to carry out, they lack sensitivity, particularly in areas of low endemicity [10],[11].

In areas of low disease transmission, where the prevalence of the infection is low, the cure for the disease after treatment needs more sensitive diagnostic techniques than searching for eggs in fecal samples as false-negative results in coproscopic investigations are sufficient to maintain the spread of the disease, even after the use of appropriate sanitary control measures [12],[13].

Technological advances in the field of parasitology have had a significant impact on the diagnosis of parasitic diseases, and molecular methods, mainly DNA amplification techniques by polymerase chain reaction (PCR) have been increasingly used [14].

The objective of this study was to compare the performance of qPCR-based techniques using fecal and serum samples, with KK and HH techniques to diagnose cases of schistosomiasis due to *S. mansoni* in the outskirts of the municipality of Barra Mansa/RJ, Brazil.

## Methods

A cross-sectional study was conducted from April to December 2011 in the districts of Siderlândia, Cantagalo, São Luiz, Nova Esperança, and Santa Clara. These localities constitute approximately 7,000 inhabitants and are located in the outskirts of Barra Mansa, Rio de Janeiro, Brazil.

The sample size was calculated assuming a prevalence of 1%. An increment of 30% was made to compensate for follow-up losses. The estimated sample size was 650 individuals residing in the neighborhoods described above. Households were systematically selected (one in six), and individuals were randomly selected by a draw among those who agreed to participate in the study. Subjects who were older than 5 years old and had not been treated for *Schistosomiasis mansoni* in the previous year were included in the study.

### Statistical analysis

Statistical analysis was performed using SPSS for Windows, version 15.0 (SPSS, Inc., Chicago IL, USA) and Microsoft Excel 2003. Significance levels of tests were fixed by accepting a type 1 error of 5% (α =0.05).

Population characteristics were described using absolute and relative frequencies and calculating the mean age and standard deviations. The proportion of positive results for each infection test (prevalence) was assessed for each diagnostic technique. Each *S. mansoni* infection measurement technique was compared and marginal associations were verified using the McNemar test [15]. Pairwise agreement between results was assessed using the Cohen kappa index (K) and 95% confidence interval.

Associations among *S. mansoni* infection and age range, sex, neighborhood, river water use, and history of schistosomiasis were assessed for each technique using the chi-square test. Fisher’s exact or likelihood ratio tests were used when the sample number was insufficient for the chi-square test. We compared the accuracy (sensitivity, specificity, likelihood ratio, and predictive values) of serological techniques to parasitological techniques, and also compared the results among techniques to determine which were most effective to diagnose *S. mansoni* in areas with a similar epidemiological profile to that of the target area of this study.

### Ethical aspects

In accordance with the Brazilian regulations on human subjects research, a written informed consent was obtained from each participant. For subjects under 18 years of age, the written informed consent was obtained from the parents or legal guardians. This project was approved by the Research Ethics Committees of the Department of Infectious and Parasitic Diseases and of Hospital das Clínicas (CAPPesq) of the School of Medicine of the University of São Paulo (approval number 0405/09).

The experimental research project followed Laws 6.638/79 and 9605/98, Decree 24.645/34, regarding the Ethical Principles of Animal Experimentation, the Principles for Research Involving Animals [16], and other guidelines that regulate animal research. The work was initiated only after the approval of the research project number CEP-IMT 2011/096 by the Ethics Committee on Animal Research of the Institute of Tropical Medicine of São Paulo, University of São Paulo, Brazil.

### Laboratory diagnosis methods

#### Specimen collection

Community health agents of the Municipal Schistosomiasis Control Program collected 572 randomized fecal and serum samples from inhabitants of 5 peripheral neighborhoods of Barra Mansa, Rio de Janeiro. Serum samples obtained were divided in aliquots and stored at −20°C, then transported to São Paulo in thermal boxes containing dry ice and stored at −20°C in the laboratory until tested. The elapsed time from collection to test was around six months.

### Parasitological methods

Fecal samples were processed and evaluated using techniques based on the technique of Kato & Miura [17] as modified by Katz *et al*. [18]. Four slides were prepared for every participant, being two slides for the KK and two for HH. A Helm Test® Kit (Biomanguinhos; Fiocruz; Rio de Janeiro; Brazil) was used to perform the KK technique.

The Kit Helm Test® Bio-Manguinhos is a qualitative-quantitative test for parasitological detection in stools. It allows the detection of all helminth eggs that are usually found in the stool: *Ascaris lumbricoides, Schistosoma mansoni*, *Hookworm*, *Trichuris*, *Taenia* and, less frequently, *Enterobius* and *Strongyloides*. The method comprises: a screen that filters the material to be examined, retaining debris that would hinder or prevent the visualization of helminth eggs; a coverslip to be pre-colored in diaphanazing fixative solution, allowing the conservation of eggs and the clearing of the smear, and a specially designed perforated plate. This implies that the same amount of feces is always examined, enabling standardization and excellent observation of a sufficient sample amount that is easily prepared and may be examined after a short time (approximately 1 hour) or stored for several months. The number of eggs of *S. mansoni* in each slide is counted through optical microscopy, multiplied by the factor 24 and the result is released in eggs per gram of feces (epg).

Major impurities were removed by passing fecal samples without preservatives through a Nylon Baltex PA-7-200/XX screen (Tecmolin, São Paulo, Brazil). A stainless steel container was used to measure approximately 500 mg samples of feces, which were subsequently stored at −20°C.

Oligonucleotide PCR primers and a TaqMan® probe were designed to target the 121-bp tandem repeat of *S. mansoni* (GenBank M61098), described by Hamburger *et al*. [19]. DNA extraction from serum, stool, and saline solutions with eggs and adult worms were performed at the Schistosomiasis Laboratory and at the Gastroenterology Laboratory, both at USP Medical School, in São Paulo, Brazil, as previously described [20],[21]. DNA from unrelated parasites (*Ascaris lumbricoides* and *Strongyloides stercoralis*) was used to evaluate the specificity of the assay. Sensitivity was determined using serial dilutions of a DNA sample extracted from 200 eggs, including 10-fold dilutions ranging from 2 to 0.002 eggs (1:10 to 1:100,000). PCR amplification efficiency was calculated to be of 103.8%, with a slope of 3.44 [21]-[23].

### DNA extraction from the serum samples

DNA extraction from the serum was performed using the guanidine isothiocyanate-phenol-chloroform (GT) method [14],[24]. DNA was stored at −20°C after extraction [23].

### DNA extraction from the fecal samples

Approximately 500 mg of stools were resuspended in 1 mL of 0.1 M PBS. Five glass beads were added to this mixture, which was homogenized for 5 Min using a vortex mixer, followed by centrifugation for 8 Min at 13,200 rpm at 4°C. An aliquot of 400 μL of the supernatant was mixed with 100 μL of a Rapid One-Step Extraction (ROSE) solution: 10 MM Tris-hydroxymethyl amino methane HCl (pH 8), 300 MM of EDTA (pH 8.0), 1% sarkosyl (sodium lauryl sarcosinate), and 1% polyvinylpolypyrrolidone (PVP) [20]. Then, 30 μL of proteinase K (Life Technologies, Carlsbad, California, USA) was added and homogenized using a vortex mixer. The sample was incubated for 120 Min at 65°C. In the second phase, DNA present in this solution was extracted and stored as described for serum samples.

### Purification of DNA extracted from fecal and serum samples

DNA extracted from serum and feces were purified using the InstaGene Matrix according to the manufacturer’s instructions. Once purified, extracted DNA was stored at −20°C until use.

### Amplification of DNA from serum and fecal samples

#### Primers and probes

DNA samples were amplified and detected using a set of primers and probes complementary to a 121-bp tandem repeat sequence of *S. mansoni* strain SM 1–7 (GenBank accession number M61098) described by Hamburger *et al*. [19]. Primer sequences were: forward F2: 5′-CCG ACC AAC CGT TCT ATG A-3′; reverse R2: 5′CAC GC TCT CGC AAA TAA TCT AAA-3′ [25]; probe PO2: 5′-6[FAM] TCG TTG TAT CTC CGA AAC CAC TGG ACG [(BHQ1])-3′, all synthesized by Sigma Life Sciences (Woodlands, Texas, USA).

All samples were evaluated using TaqMan® Reagents Exogenous Internal Positive Control (IPC) (Life Technologies), according to the manufacturer’s instructions to check for the presence of *Taq* DNA polymerase inhibitors.

### TaqMan® Real-Time conditions of serum and fecal samples

TaqMan® Real-Time PCR was performed in a final volume of 20 μL containing: 10 μL of TaqMan® Universal PCR Master Mix 2×; 20 pmol of primers F2 and R2, 5 pmol of the FAM-labelled PO2 probe (Sigma Custom Products), and 2 μL of purified DNA. For each sample, another reaction was performed in parallel using the TaqMan® Reagents Exogenous Internal Positive Control (IPC) in a final volume of 21 μL, containing the following: 10 μL of TaqMan® Universal PCR Master Mix 2×, 5 μL of 10× exogenous IPC mix, 1 μL of 50× Exo IPC, and 5 μL of the purified DNA sample. For each batch of reactions, two other controls were used: no amplification control (NAC) and no template control (NTC). PCR was performed in an Applied Biosystems 7300 Real-Time PCR System® (Life Technologies) using the following cycling conditions: 50°C for 2 Min, 95°C for 10 Min, and 40 cycles at 95°C for 15 s and 60°C for 1 Min. The amplification reaction was performed in an Applied Biosystems 7300 Real-Time PCR System® using the following amplification program: 50°C for 2 Min; 95°C for 10 Min; 40 cycles at 95°C for 15 s, and 60°C for 1 Min.

For each batch of reactions, two positive controls were used for qPCR-feces and qPCR-serum, respectively: DNA extracted from human feces marked with 0.9% saline solution containing 200 *S. mansoni* eggs and DNA extracted from saline 0.9% *S. mansoni* containing 200 eggs, employing the same standardization [23].

To minimize the possibility of contamination, DNA extraction and DNA amplification protocols were performed in distinct rooms. All experiments were performed inside positive pressure air-flow cabinet, using frequent UV irradiation, and employing only disposable sterile laboratory equipment including pipette tips with filters.

### Criteria used to evaluate the results obtained by TaqMan® Real-Time PCR using stool and serum samples

**Figure 1:**
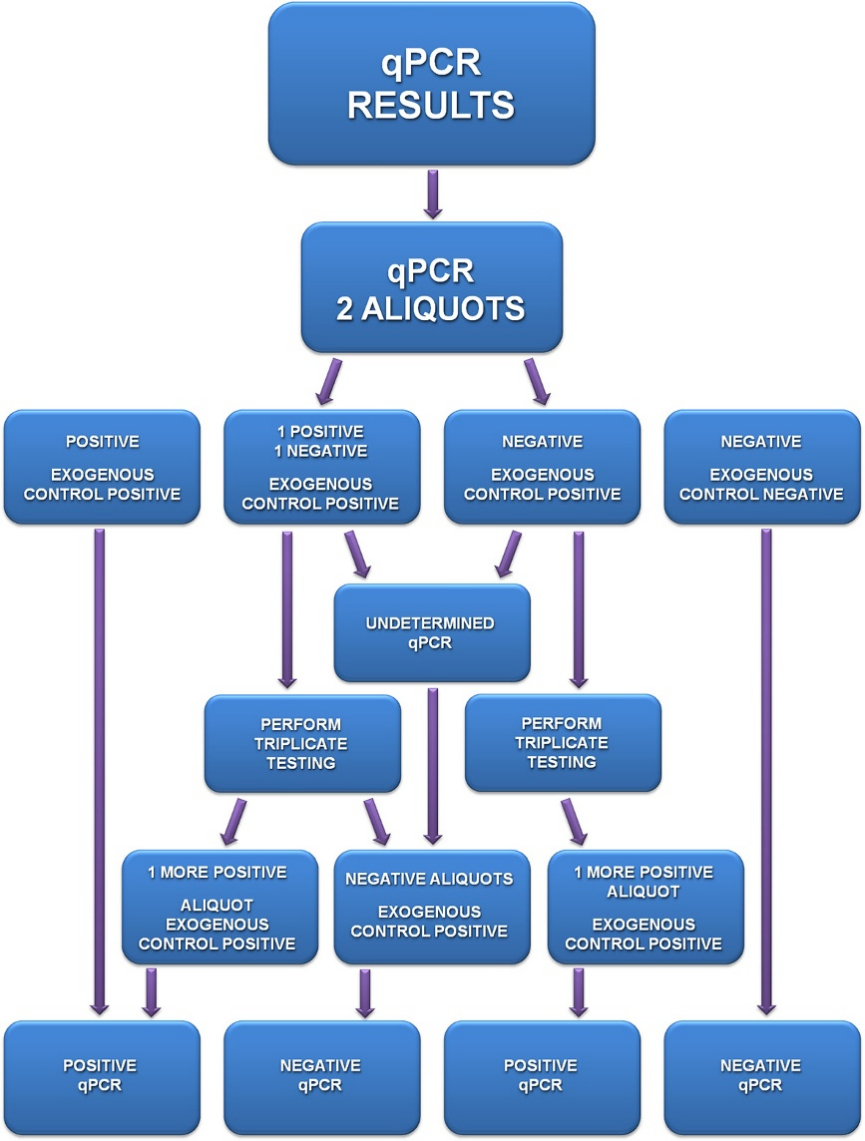
**Algorithm of the criteria used to evaluate the results obtained by the TaqMan® Real-Time PCR technique using fecal and serum samples.**

The criteria used to evaluate the qPCR were as follows (Figure 1):

Positive qPCR: Duplicates of the testing sample amplified by qPCR;

Undetermined qPCR: One aliquot of the testing sample was amplified by qPCR;

qPCR IPC: Exogenous control for the testing sample was not amplified (negative) by qPCR.

The undetermined qPCR and qPCR IPC samples were tested in triplicate. Duplicate amplifications of the samples obtained by qPCR were included in the positive results.

## Results

A total of 650 individuals agreed to participate in this study. The following techniques were performed for each participant: qPCR-feces, qPCR-serum, KK, and HH. The socio-demographic characteristics of the sampled population were also analyzed.

A total of 572 paired serum and fecal samples from volunteers were assessed using qPCR-serum and stool techniques and the KK and HH parasitological techniques.

Most subjects in the study population were females, with a mean age of 40–41 years. Siderlândia neighborhood had the highest number of subjects (243/42.5%), whereas Santa Clara had the smallest number of subjects (32/5.6%). Approximately 4.2% of the participants reported a history of schistosomiasis.

The qPCR-feces identified *S. mansoni* infections in 9.6% (55/572) samples, followed by qPCR-serum (1.4%; 8/572). The parasitological techniques (KK-HH) identified the lowest number of infections (0.9%; 5/572) (Table 1). Table 2 shows the characteristics of the five subjects with positive results for the KK and/or HH techniques. The qPCR over feces-derived DNA was able to detect four positive results regarding the parasitological techniques, while the qPCR technique-serum was positive in only 1/5 positive individuals in the parasitological techniques.

**Table 1:** ***Schistosoma mansoni*** **-positive infections for each diagnostic technique in samples collected from the sampled population in Barra Mansa/RJ - 2011**

Technique	Positive/Total	%
KK-HH	5/572	0.9
qPCR-feces	55/572	9.6
qPCR-serum	8/572	1.4

*KK*, Kato-Katz technique.

*HH*, Hoffman technique.

qPCR-feces-qPCR-feces technique.

qPCR-serum-qPCR-serum technique.

**Table 2:** **Associations between parasitological tests positive for** ***Schistosoma mansoni*** **infection and molecular techniques of the sampled population in the municipality of Barra Mansa/RJ - 2011**

Sample	A	Sex	KK S1	KK S2	HH S1	HH S2	qPCR Feces	qPCR serum
J.B.S.	46	M	1 egg	0	SM	SM	+	-
M.C.	52	M	8 eggs	19 eggs	SM	SM	+	-
I.R.S.	42	M	6 eggs	2 eggs	SM	SM	+	+
L.B.A.	21	M	6 eggs	1egg	-	SM	+	-
C.F.S.	19	F	0	0	SM	0	-	-

*A*, Age.

*KK*, Kato-Katz technique.

*HH*, Hoffman technique.

*S1*, first slide; *S2*, second slide.

qPCR-feces- qPCR-feces technique.

qPCR-serum- qPCR-serum technique.

*SM*, positive for *Schistosoma mansoni.*

When compared to the parasitological techniques, the qPCR-feces technique was able to detect four positive results, whereas the qPCR-serum technique was positive for only one out of five subjects who showed positive results in the parasitological techniques. The qPCR-feces technique showed higher sensitivity when compared to the parasitological techniques, followed by the qPCR-serum technique (Table 3). Table 4 and Graphic 1 show that the positivity rate obtained from positive and undetermined qPCR tests occurred at 9.6% and 8.7%, and with medians of 34.8 and 37.1, respectively. Table 5 and Graphic 2 show that the Threshold Cycle (Ct) values of positive and undetermined qPCR-serum tests were 1.4% (n =8) and 5.4% (n =31) with medians of 36.3 and 37.0, respectively (Additional files 1 and 2).

**Table 3:** **Rates of sensitivity, specificity, positive predictive value, and negative predictive value of the diagnostic techniques compared to the parasitological techniques**

Method	Measurement	Estimate (%)	CI 95%minimum	CI 95%maximum
qPCR-feces	Sensitivity	80.0	28.4	99.5
	Specificity	92.4	90.0	94.4
	Positive Predictive Value (PPV)	8.0	2.2	19.2
	Negative Predictive Value(NPV)	99.8	99.0	100.0
qPCR-serum	Sensitivity	20.0	0.5	71.6
	Specificity	98.8	97.5	99.5
	Positive Predictive Value (PPV)	12.5	0.3	52.7
	Negative Predictive Value (NPV)	99.3	98.2	99.8

**Table 4:** **Results from positive and undetermined qPCR tests from fecal samples along with Ct values obtained from the sampled population in the municipality of Barra Mansa/RJ - 2011**

Method	Cases/Total	%	Ct values (Log) qPCR				
			Mean	SD	Median	Minimum	Maximum
Positive qPCR-feces	55/572	9.6	33.6	4.9	34.8	14.7	38.8
Undetermined qPCR-feces	50/572	8.7	32.3	8.5	37.1	11.5	40

**Table 5:** **Results from positive and undetermined qPCR tests from serum samples along with Ct values obtained from the sampled population in the municipality of Barra Mansa/RJ - 2011**

Method	Cases/Total	%	Ct values (Log) qPCR				
			Mean	SD	Median	Minimum	Maximum
Positive qPCR-serum	8/572	1.4	36.9	1.3	36.3	38.8	35.2
Undetermined qPCR-serum	31/572	5.4	35.5	4.4	37	39.7	20.3

Table 6 shows that positivity rates obtained from the qPCR-feces technique was significantly higher than that obtained from the parasitological techniques (p <0.001). No statistically significant differences were observed when the techniques qPCR-serum and HH and KK were compared.

**Table 6:** **Agreement among the results obtained by the techniques KK and HH and those obtained by qPCR-serum and qPCR-feces, in fecal and serum samples collected from the sampled population in the municipality of Barra Mansa/RJ - 2011**

Test	KK-HH				Total		p McNemar	Kappa	CI (95%)	
	Negative		Positive							
	N	%	n	%	n	%			Minimum	Maximum
qPCR-feces										
Negative	549	(90)	1	(0.2)	550	90.2	<0.001	0.110	0.008	0.212
Positive	56	(9.2)	4	(0.7)	60	9.8				
Total	605	(99.2)	5	(0.8)	610	100				
qPCR-serum										
Negative	560	(97.9)	4	(0.7)	564	98.6	0.549	0.145	−0.123	0.412
Positive	7	(1.2)	1	(0.2)	8	1.4				
Total	567	(99.1)	5	(0.9)	572	100				

There was a poor agreement among the qPCR and parasitological techniques.

Table 6 shows that the positivity rate obtained from the qPCR-feces technique was statistically higher than that obtained from the parasitological techniques (p <0.001). No statistically significant differences were observed when the techniques qPCR-serum and HH and KK were compared. There was a poor agreement among the qPCR and parasitological techniques.

## Discussion

Laboratory diagnosis of *S. mansoni* infection is an important step for the identification of new cases, early treatment, surveillance, and control of this helminthic infections in areas of low endemicity and in the presence of infections with low parasite load (<100 epg) [12].

In areas of low endemicity, the use of parasitological techniques to diagnose schistosomiasis due to *S. mansoni* shows limited sensitivity. In the past decade, diagnostic methods based on the detection of antigens and antibodies have been developed; nevertheless, these techniques have presented limitations regarding to sensitivity and specificity due to cross-reactions with other parasites, often leading to false-positive results [26],[27].

Recent technological advancements in the field of parasitology have had a significant impact in the diagnosis of parasitic diseases, and molecular methods, mainly DNA amplification techniques by PCR, have been increasingly used [14].

In *S. mansoni*, the gene cluster encoding for the ribosomal RNA (rRNA) represented the first described region of repetitive DNA. This complex is composed of tandem repeat DNA units. Each unit contains approximately 10 kb that encodes highly conserved types of eukaryotic rRNA, 5.8S, 18S, and 28S [28],[29].

A new diagnostic technique based on the use of PCR was developed. This technique has been shown to be sensitive and specific to detect infections in snails and to identify cercariae in water bodies [19]. The first region of repetitive DNA described in *S. mansoni* comprised the ribosomal RNA (rRNA) gene cluster [28],[29], similar to hypervariable regions of the human genome [30].

Research has been conducted to develop molecular techniques using different types of samples to diagnose this parasitic disease [31]-[33]. The first study performed with fecal and serum samples revealed that conventional PCR could detect 2.4 fg of *S. mansoni* DNA/gram of feces. This assay was shown to be twice as sensitive as the KK method [25].

In this project, we developed a TaqMan® Real-Time PCR assay, using an internal positive control (IPC, Life Technologies) for the presence of inhibitors. The qPCR-feces technique presented positivity rates of 9.6% (n =55/572), which were 12 times higher than the KK and HH parasitological techniques (positivity rate of 0.9%; n =5/572). The qPCR-serum technique demonstrated a positivity rate of 1.5% (n =8/572), being approximately twice that of the parasitological techniques. Statistically significant differences were observed, with a greater significance observed for the qPCR-feces technique (p <0.05).

A limitation of this study was the feces collection on a single day per individual. The sensitivity could have been improved, as in the case of the KK test, by collecting feces on two or three consecutive days, followed by testing, to meet daily egg production variability in an infected individual. However, this was not done here, mainly because of the high number of individuals enrolled in our study.

In this study, the qPCR-feces reaction was able to detect four out of five positive cases for *S. mansoni* by the KK and HH techniques. The cycle threshold value (Ct value) of the positive fecal samples showed a median of 34.8. The individual, who did not present positive results in the qPCR-feces assay, presented negative results to the KK technique. Hove *et al.*[34] reported a decrease in agreement and sensitivity of Multiplex q-PCR when compared to KK, for infections with low parasite load (less than 100 epg). Pontes *et al*. [35] and Gomes *et al*. [36] obtained false-negative results when performing conventional PCR, and attributed these results to the presence of inhibitors of the amplification reaction in fecal samples, variations in the distribution and release of *S. mansoni* eggs in the feces [37],[38] or to DNA degradation during transportation of the sample from the endemic area to the laboratory. In our case, the possible inhibition of the reaction had to be discarded because the exogenous control of the qPCR-feces reaction was positive.

There was one individual who had positive results for the techniques HH, KK, qPCR-feces, and qPCR-serum, with low parasite load. The positivity rates of qPCR-serum were higher than those observed in the parasitological techniques, but lower than those observed in the other techniques, including the qPCR-feces. Pontes *et al*. [35] also observed a lower sensitivity of conventional PCR-serum in comparison to PCR-feces to detect *S. mansoni* infection, when compared to the KK technique. Nevertheless, according to Wichmann *et al*. [32] and Zhou *et al*. [39], positive serum reactions are related to the acute phase of the schistosoma infection where the inflammatory response typical of this phase leads to the circulation of higher levels of degradation products of schistosomula, adult worms, and eggs, causing an increase in circulating DNA, particularly up to the eighth week post-infection. This would explain the DNA amplification observed in the qPCR-serum technique.

The medians of Ct values of the logarithmic curves obtained from qPCR-feces and qPCR-serum were 34.8 and 36.3, respectively, indicating a low amount of amplifiable DNA template, equivalent to about 0.02 eggs (at the 1/10,000 dilution), as observed in the standard curve. This fact could be related to the low parasite load typical of *S. mansoni* infections in areas of low endemicity [34].

The exogenous control system TaqMan® Exogenous Internal Positive Control Reagents (IPC) was used to evaluate the quality of the extracted DNA and the presence of inhibitors of the qPCR technique in serum and fecal samples.

Several studies have emphasized the complexity of using fecal samples in PCR techniques [25],[35],[36],[40]. In our study, despite all the care taken during DNA extraction and purification, an inhibition of the exogenous control amplification was still observed in 16.2% of the fecal samples. Positive exogenous controls were observed in all samples of serum subjected to the same treatment. The group of IPC-negative samples may also include false-negative results. However, after repeating the qPCR technique for samples in this group, positive results for *S. mansoni* DNA detection were not observed and the exogenous controls remained negative.

Inhibition of amplification of the exogenous control was not observed for the qPCR-serum technique. Duplicates of amplified *S. mansoni* DNA were considered positive for up to 40 cycles of the TaqMan® qPCR assay. However, a significant number of undetermined results (31/612) were observed for the qPCR-serum technique, with a median Ct value of 37.

Taking into account that this is an intravascular helminthic infection, where a high Ct value indicates a low quantity of *S. mansoni* template DNA, in terms of epidemiological analysis, what would be considered as a qPCR positive result in fecal and serum samples?

When considering the KK technique, which is used to control this endemic disease, a positive test result observed after systematic repetition of a negative test would classify the individual as positive for *S. mansoni* infection and this individual would then be treated with a specific chemotherapy [10],[41].

Although positivity has been defined in the present study as the presence of amplified *S. mansoni* DNA in duplicate tests, if one adopts the same criteria as that used for the parasitological technique, the presence of amplified *S. mansoni* DNA in one or two reactions would be considered as a positive result.

Wichmann *et al*. [32],[42] reported a higher percentage of positive results in real-time PCR using serum from patients with acute schistosomiasis, where positivity was defined as the presence of amplified *S. mansoni* DNA in duplicate tests, with a limit of Ct <45.

Despite the factors involved in the inhibition of the qPCR-feces technique and the low parasite load of the *S. mansoni* infection observed in the sampled population, qPCR-feces positive results were 6.8-fold higher (55/572) than the KK and HH techniques (5/572). The positivity rate of the qPCR-serum technique (8/572) was approximately 1.6-fold higher than that observed for the parasitological techniques, in an area of very low endemicity.

According to Cnops *et al.*[43], all PCR-based techniques to detect genomic DNA of *S. mansoni* were able to detect the DNA from all phases of the life cycle of this parasite: eggs containing miracidia, cercariae, schistosomulae, and adult worms. In summary, a positive PCR assay indicates the presence of the parasite but does not provide information regarding its life cycle phase or its viability, including the presence of mature, male and female worms capable of egg deposition. This is likely to explain why we have not found a positive correlation between the Ct value and the number of eggs.

The technique of Kato & Miura [17] as modified by Katz *et al.*[18] is internationally recognized as the standard method to diagnose schistosomiasis [2]. However, this technique has shown a lower sensitivity in individuals with a low parasite load, in areas of low endemicity, and receiving a particular therapy [44],[45], which compromises the evaluation of the accuracy of new techniques to diagnose *S. mansoni* infections, when compared to the standard technique, which was used as reference. Therefore, the TaqMan® qPCR technique represents a potential diagnostic tool for non-invasive *S. mansoni* infections, including those with a low parasite load.

It is well known that the parasitological techniques, particularly the Kato-Katz technique, have significantly contributed to the control of this parasitic infection. We consider areas of low endemicity as indicators of the positive results obtained by the Brazilian National Programs for Schistosomiasis Control. However, areas of low endemicity and transmission of schistosomiasis without adequate surveillance may potentially change into areas of high endemicity [13],[46]. Thus, it is necessary to develop strategies to control this helminthic disease in areas of low endemicity, so that surveillance measures equal to those used in other areas may be applied to populations from these localities, leading to the eradication of schistosomiasis.

## Conclusions

This study offers a perspective that indicates the possibility of combining these diagnostic tools to improve the diagnosis of schistosomiasis in infections of low parasite load, not only in the clinical setting, but also in epidemiological studies.

## Additional files

## Electronic supplementary material

Additional file 1: Median “Threshold Cycle” (Ct) values as obtained for positive and undetermined qPCR tests in feces samples obtained from the sampled population in the municipality of Barra Mansa/RJ - 2011.(TIF 52 KB)

Additional file 2: Median “Threshold Cycle” (Ct) values as obtained for positive and undetermined qPCR tests in fecal samples obtained from the sampled population in the municipality of Barra Mansa/RJ - 2011.(TIF 33 KB)

Below are the links to the authors’ original submitted files for images.

Authors’ original file for figure 1

## References

[1] Chitsulo L, Engels D, Montresor A, Savioli L (2000). The global status of schistosomiasis and its control. Acta Trop.

[2] Media Centre. Schistosomiasis. Fact sheet no 115. 2012, World Health Organization, Geneva

[3] Sistema de Informação do Programa de Controle da Esquistossomose. Sistema de Informação de Agravos em Saúde/2012. Casos confirmados de Esquistossomose, 1995 a 2011. Brasília (DF). 2012, Grandes Regiões e Unidades Federadas, Brasil

[4] Bergquist NR (2002). Schistosomiasis: from risk assessment to control. Trends Parasitol.

[5] Pordeus LC, Aguiar LR, Quinino LRM, Barbosa CS (2008). A ocorrência das formas aguda e crônica da esquistossomose mansônica no Brasil no período de 1997 a 2006: uma reviSão de literatura. Epidemiol Serv Saúde.

[6] Report of Informal Consultation on Schistosomiasis in low Transmission Areas: Control Strategies and Criteria for Elimination, London, 10-13 April 2000. 2001, World Health Organization, Geneva

[7] de Janeiro R (2009). Secretaria de Estado de Saúde e Defesa Civil.

[8] Coordenadoria de Epidemiologia. 2008, Documentos retirados do SINANNET/07-05-2008, Barra Mansa. Rio de Janeiro

[9] Conselho de Desenvolvimento Social. 1976, Programa Especial de Controle da Esquistossomose no Brasil, Brasília (DF)

[10] Ruiz-Tiben E, Hillyer GV, Knight WB, De Rios GI, Woodall JP (1979). Intensity of infection with *Schistosoma mansoni*: its relationship to the sensitivity and specificity of serologic tests. Am J Trop Med Hyg.

[11] Ebrahim A, El-Morshedy H, Omer E, El-Daly S, Barakat R (1997). Evaluation of the Kato-Katz thick smear and formol ether sedimentation techniques for quantitative diagnosis of *Schistosoma mansoni*infection. Am J Trop Med Hyg.

[12] Cavalcanti MG, Silva LF, Peralta RH, Barreto MG, Peralta JM (2013). Schistosomiasis in areas of low endemicity: a new era in diagnosis. Trends Parasitol.

[13] de Noya BAA, Guevara RR, Colmenares C, Losada S, Noya O (2006). Low transmission areas of schistosomiasis in Venezuela: consequences on the diagnosis, treatment, and control. Mem Inst Oswaldo Cruz.

[14] Saiki R, Gelfand D, Stoffel S, Scharf S, Higuchi R, Horn G, Mullis K, Erlich H (1988). *Primer*-directed enzymatic amplification of DNA with a thermostable DNA polymerase. Science.

[15] Kirkwood B, Sterne JAC (2003). Essential Medical Statistics.

[16] HSMO. Her Majesty's Stationery Office. Office of Public Sector Information (OPSI): *Geneva Conventions (Amendment) Act 1995 (c. 27).* London; 1995.

[17] Kato K, Miura M (1954). Comparative examinations. Jpn J Parasitol.

[18] Katz N, Chaves A, Pellegrino J (1972). A simple device for quantitative stool thick-smear technique in Schistosomiasis mansoni. Rev Inst Med Trop Sao Paulo.

[19] Hamburger J, Turetski T, Kapeller I, Deresiewicz R (1991). Highly repeated short DNA sequences in the genome of *Schistosoma mansoni*recognized by a species-specific probe. Mol Biochem Parasitol.

[20] Espírito-Santo MCC, Alvarado-Mora MV, Pinto PL, Botelho LS, Dias-Neto E, Chieffi PP, Carrilho FJ, Pinho JR, Gryschek RCB: Early Detection of Schistosoma Mansoni Infection by Real Time PCR in Hamster Model - is a Serum Sample Better Than Feces? In *Em: XVIII International Congress for Tropical Medicine and Malaria, 2012, Rio de Janeiro.* Anais, v. II; 2012:662-663.

[21] Espírito-Santo MCC, Alvarado-Mora MV, Pinto PL, Carrilho FJ, Pinho JR, Gryschek RCB (2012). Two sequential PCR amplifications for detection of *Schistosoma mansoni*in fecal samples with low parasite load. Rev Inst Med Trop Sao Paulo.

[22] Espírito-Santo MCC, Alvarado-Mora MV, Pinto PLS, Carrilho FJ, Pinho JR, Gryschek RCB (2011). Padronização de métodos de extração de DNA para a detecção do Schistosoma mansoni para áreas de baixa endemicidade. XLVII Congresso da Sociedade Brasileira de Medicina Tropical; 2011, 85.

[23] Espírito-Santo MC, Alvarado-Mora MV, Pinto PL, de Brito T, Botelho-Lima L, Heath AR, Amorim MG, Dias-Neto E, Chieffi PP, Pinho JR, Carrilho FJ, Albuquerque Luna EJ, Gryschek RC (2014). Detection of *Schistosoma mansoni*infection by TaqMan® Real-Time PCR in a hamster model. Exp Parasitol.

[24] Chomczynski P, Sacchi N (1987). Single-step method of RNA isolation by acid guanidinium thiocyanate-phenol-chloroform extraction. Anal Biochem.

[25] Pontes LA, Dias-Neto E, Rabello A (2002). Detection by polymerase chain reaction of *Schistosoma mansoni*DNA in human serum and feces. Am J Trop Med Hyg.

[26] Doenhoff MJ, Chiodini PL, Hamilton JV (2004). Specific and sensitive diagnosis of schistosome infection: can it be done with antibodies?. Trends Parasitol.

[27] Sorgho H, Bahgat M, Poda JN, Song W, Kirsten C, Doenhoff MJ, Zongo I, Ouedraogo JB, Ruppel A (2005). Serodiagnosis of *Schistosoma mansoni*infections in an endemic area of Burkina Faso: performance of several immunological tests with different parasite antigens. Acta Trop.

[28] Simpson AJ, Dame JB, Lewis FA, McCutchan TF (1984). The arrangement of ribosomal RNA genes in *Schistosoma mansoni*. Identification of polymorphic structural variants. Eur J Biochem.

[29] van Keulen H, Loverde PT, Bobek LA, Rekosh DM (1985). Organization of the ribosomal RNA genes in *Schistosoma mansoni*. Mol Biochem Parasitol.

[30] Spotila LD, Rekosh DM, LoVerde PT (1991). Polymorphic repeated DNA element in the genome of *Schistosoma mansoni*. Mol Biochem Parasitol.

[31] Enk MJ, Lustosa Lima AC, Drummond SC, Schall VT, Coelho PMZ (2008). The impact of the number of fecal samples on the prevalence, the infection intensity and the distribution of the infection with *Schistosoma mansoni*among a population in an area of low transmission. Acta Trop.

[32] Wichmann D, Poppert S, Thien HV, Clerinx J, Dieckmann S, Jensenius M, Philippe Parola GD, Richter J, Schunk M, Stich A, Zanger P, Burchard GD, Egbert Tannich E (2013). Prospective European-wide multicentre study on a blood based real-time PCR for the diagnosis of acute schistosomiasis. BMC Infect Dis.

[33] Gomes AL, Melo FL, Werkhauser RP, Abath FG (2006). Development of a real time polymerase chain reaction for quantitation of *Schistosoma mansoni*DNA. Mem Inst Oswaldo Cruz.

[34] ten Hove RJ, Verweij JJ, Vereecken K, Polman K, Dieye L, van Lieshout L (2008). Multiplex real-time PCR for the detection and quantification of *Schistosoma mansoni* and *S. haematobium*infection in fecal samples collected in northern Senegal. Trans R Soc Trop Med Hyg.

[35] Pontes LA, Oliveira MC, Katz N, Dias-Neto E, Rabello A (2003). Comparison of a polymerase chain reaction and the Kato-Katz technique for diagnosing infection with *Schistosoma mansoni*. Am J Trop Med Hyg.

[36] Gomes LI, Marques LH, Enk MJ, Coelho PM, Rabello A (2009). Further evaluation of an updated PCR assay for the detection of *Schistosoma mansoni*DNA in human fecal samples. Mem Inst Oswaldo Cruz.

[37] Teesdale CH, Fahringer K, Chistulo L (1985). Egg count variation and sensitivity of a thin smear technique for the diagnosis of *Schistosoma mansoni*. Trans R Soc Trop Med Hyg.

[38] Engels D, Sinzinkayo E, Gryseels B (1996). Day-to-day egg count fluctuation in *Schistosoma mansoni*infection and its operational implications. Am J Trop Med Hyg.

[39] Zhou L, Tang J, Zhao Y, Gong R, Lu X, Gong L, Wang Y (2011). A highly sensitive TaqMan real-time PCR assay for early detection of Schistosoma species. Acta Trop.

[40] Oliveira LM, Santos HL, Gonçalves MM, Barreto MG, Peralta JM (2010). Evaluation of polymerase chain reaction as an additional tool for the diagnosis of low-intensity *Schistosoma mansoni*infection. Diagn Microbiol Infect Dis.

[41] Carneiro TR, Pinheiro MC, de Oliveira SM, Hanemann AL, Queiroz JA, Bezerra FS (2012). Increased detection of schistosomiasis with Kato-Katz and SWAP-IgG-ELISA in a Northeastern Brazil low-intensity transmission area. Rev Soc Bras Med Trop.

[42] Wichmann D, Panning M, Quack T, Kramme S, Burchard GD, Grevelding C, Drosten C (2009). Diagnosing schistosomiasis by detection of cell-free parasite DNA in human plasma. PLoS Negl Trop Dis.

[43] Cnops L, Tannich E, Polman K, Clerinx J, Van Esbroeck M (2012). Schistosoma real-time PCR as diagnostic tool for international travellers and migrants. Trop Med Int Health.

[44] Enk MJ, Oliveira E, Silva G, Rodrigues NB (2012). Diagnostic accuracy and applicability of a PCR system for the detection of *Schistosoma mansoni*DNA in human urine samples from an endemic area. PLoS One.

[45] Zhang YY, Luo JP, Liu YM, Wang QZ, Chen JH, Xu MX, Xu JM, Wu J, Tu XM, Wu GL, Zhang ZS, Wu HW (2009). Evaluation of Kato-Katz examination method in three areas with low-level endemicity of schistosomiasis japonica in China: a Bayesian modeling approach. Acta Trop.

[46] Estudo parasitológico da transmisSão e dos impactos da profilaxia da esquistossomose mansônica no municípioa de Bananal. 2001, Brasil. Tese. Universidade Estadual de Campinas, Instituto de Biologia, Estado de São Paulo

