# Supplementary figures and images for: Evaluation of real-time PCR assay to detect Schistosoma mansoni infections in a low endemic setting
Source: BMC Infect Dis. 2014 Oct 23;14:558. doi: 10.1186/s12879-014-0558-4 (PMC4210485; doi:10.1186/s12879-014-0558-4)

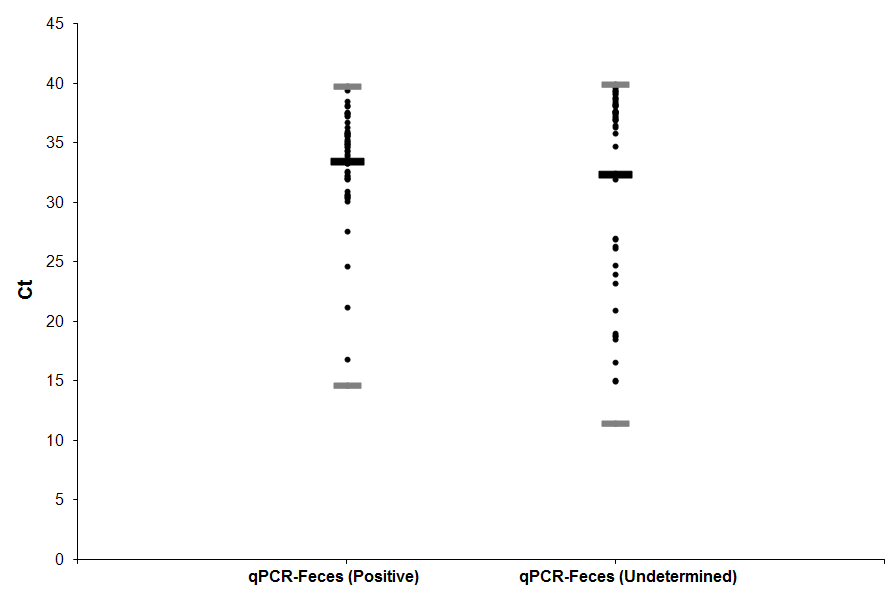

Supplement: Supplementary file 1 — Additional file 1: Median “Threshold Cycle” (Ct) values as obtained for positive and undetermined qPCR tests in feces samples obtained from the sampled population in the municipality of Barra Mansa/RJ - 2011.(TIF 52 KB) [file 12879_2014_558_MOESM1_ESM.tif]

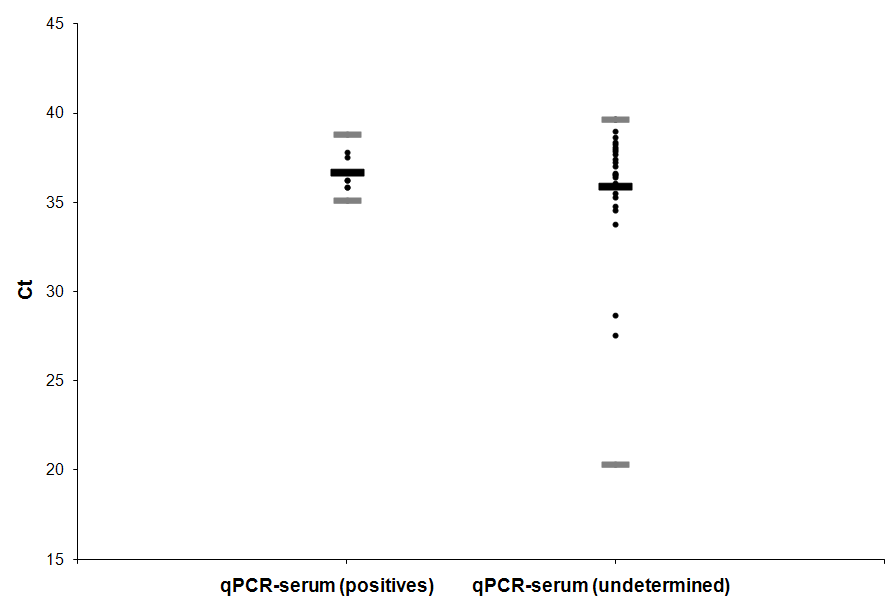

Supplement: Supplementary file 2 — Additional file 2: Median “Threshold Cycle” (Ct) values as obtained for positive and undetermined qPCR tests in fecal samples obtained from the sampled population in the municipality of Barra Mansa/RJ - 2011.(TIF 33 KB) [file 12879_2014_558_MOESM2_ESM.tif]

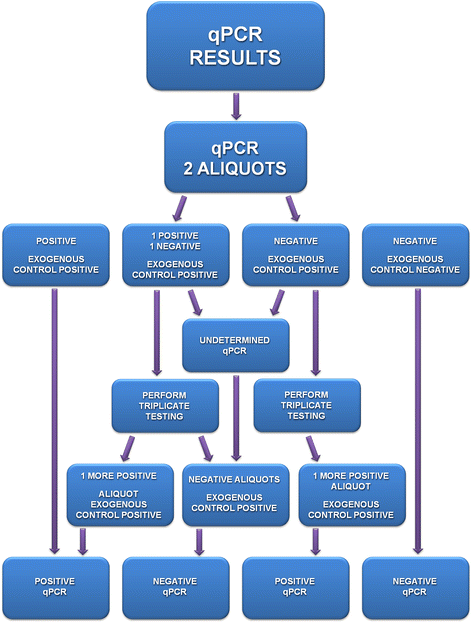

Supplement: Supplementary file 3 — Authors’ original file for figure 1 [file 12879_2014_558_MOESM3_ESM.gif]
